# What influenced people with chronic or refractory breathlessness and advanced disease to take part and remain in a drug trial? A qualitative study

**DOI:** 10.1186/s13063-020-4129-2

**Published:** 2020-02-22

**Authors:** N. Lovell, S. N. Etkind, S. Bajwah, M. Maddocks, I. J. Higginson

**Affiliations:** Cicely Saunders Institute of Palliative Care, Policy and Rehabilitation, King’s College London, Bessemer Road, London, SE5 9PJ UK

**Keywords:** Qualitative, Randomised controlled trial, Palliative care, Breathlessness, Recruitment, Retention, Person-centred care

## Abstract

**Background:**

Recruitment and retention in clinical trials remains an important challenge, particularly in the context of advanced disease. It is important to understand what affects retention to improve trial quality, minimise attrition and reduce missing data. We conducted a qualitative study embedded within a randomised feasibility trial and explored what influenced people to take part and remain in the trial.

**Methods:**

We conducted a qualitative study embedded within a double-blind randomised trial (BETTER-B[Feasibility]: BETter TreatmEnts for Refractory Breathlessness) designed using a person-centred approach. Participants with cancer, chronic obstructive pulmonary disease (COPD), interstitial lung disease (ILD), or chronic heart failure (CHF), with a modified Medical Research Council dyspnoea scale grade of 3/4 were recruited from three UK sites. A convenience subsample completed qualitative interviews after the trial. Interviews were analysed using thematic analysis. Results were considered in relation to the core elements of person-centred care and our model of the person-centred trial.

**Results:**

In the feasibility trial 409 people were screened for eligibility, and 64 were randomised. No participant was lost to follow-up. Twenty-two participants took part in a qualitative interview. Eleven had a diagnosis of COPD, 8 ILD, 2 CHF and 1 lung cancer. The participants’ median age was 71 years (range 56–84). Sixteen were male. Twenty had completed the trial, and two withdrew due to adverse effects. The relationship between patient and professional, potential for benefit, trial processes and the intervention all influenced the decision to participate in the trial. The relationship with the research team and continuity, perceived benefit, and aspects relating to trial processes and the intervention influenced the decision to remain in the trial.

**Conclusions:**

In this feasibility trial recruitment targets were met, attrition levels were low, and aspects of the person-centred approach were viewed positively by trial participants. Prioritisation of the relationship between the patient and professional; person-centred processes, including home visits, assistance with questionnaires, and involvement of the carer; and enabling people to participate by having processes in line with individual capabilities appear to support recruitment and retention in clinical trials in advanced disease. We recommend the integration of a person-centred approach in all clinical trials.

**Trial registration:**

ISRCTN Registry, ISRCTN32236160. Registered on 13 June 2016.

## Background

Recruitment and retention in clinical trials remains an important challenge which can impact the validity of results by introducing bias and reducing power. Of 151 randomised controlled trials (RCTs) funded and published by the UK’s National Institute for Health Research (NIHR), the target sample size was only achieved in 56% [1]. Recruitment to clinical trials in people with advanced disease or in palliative care can be particularly challenging. For example, a recent systematic review found the target sample size was only achieved in 36.8% of trials assessing a therapeutic intervention [2]. Eligibility can be a major limiting factor affecting recruitment in advanced disease. Trials often need to screen 10–15 patients to recruit 1, and strategies to improve recruitment have had variable success [3–11]. To advance the evidence base in palliative care we need high-quality clinical trials, including Clinical Trials of an Investigational Medicinal Product, of which there are few, in part due to these challenges [12].

Retention in clinical trials is perhaps even more important and has recently been identified as a top priority [13–15], with high levels of attrition a well-recognised problem. A review of clinical trials in advanced cancer identified a median attrition of 26% at the primary end point, increasing to 44% at the end of the study [16]. Reasons for attrition included a high symptom burden (21%), patient preference (15%), hospitalisation (10%), and death (6%) [16]. Attrition can lead to high levels of missing data, the level of which, in a recent systematic review of palliative care trials, was associated with study duration and an increasing number of study questionnaires and/or tests [17]. However, even for palliative care drug trials of short duration (4 weeks), attrition has been shown to be high, with only 40% of participants achieving the primary end point in a trial of pregabalin for cancer-induced bone pain [5]. A review of 108 RCTs of palliative care interventions found that the reason for missing data was unclassified in 53%, recorded as loss to follow-up or withdrawal with no further details of the underlying reason [18]. Meta-ethnographic review has identified five themes which may influence nonretention in trials:

1. Aspects of the trial did not fit with sense of self.
2. The trial design was not individualised.
3. Trial processes were not in line with individual capabilities.
4. Concerns about the trial medication
5. The extent to which trial participation could be appropriately accommodated to individuals’ broader lives [19]

Research within clinical trials units has considered methods that may improve recruitment and retention, identifying the importance of support and training for researchers and clinicians and choice of appropriate outcome measures [14, 20]. However, strategies to improve recruitment into trials have had variable success [3]. There is an increasing literature on person-centeredness in trials, with growing evidence that involving patients at the research design stage can direct recruitment and retention strategies and improve enrolment [21–23]. Patient and public involvement (PPI) is one method of applying person-centeredness to trials and can help to ensure that the research process is participant-friendly and trial information is relevant, readable and understandable [24, 25]. While studies evaluating person-centred care in trials remain limited, Chhatre et al. applied a conceptual model of patient-centred recruitment and retention to an RCT of patients with newly diagnosed prostate cancer [26]. The study identified strategies which may aid recruitment and retention. However, limitations due to time and resource constraints were acknowledged, and attrition was 26% at one of the three sites [26].

As more people approach the end of their lives with chronic and complex conditions, the need for robust research and evidence has never been greater. However, clinical trials in palliative care remain sparse, often limited by poor funding and methodological weaknesses [2, 27, 28]. It is therefore important to understand what affects retention so that we can minimise attrition and ensure high-quality clinical trials of palliative care interventions in the future. We conducted a qualitative study embedded within a randomised feasibility designed using a person-centred approach. The study aimed to explore what influenced participants to take part and remain in the trial.

## Methods

### Design

We conducted a qualitative study embedded within a randomised trial of mirtazapine for chronic or refractory breathlessness (BETTER-B[Feasibility]: BETter TreatmEnts for Refractory Breathlessness). The trial design aimed to optimise recruitment and retention through the use of a person-centred approach, which has been shown to enable engagement and improve patient outcomes in advanced disease [29–31].

**Fig. 1:**
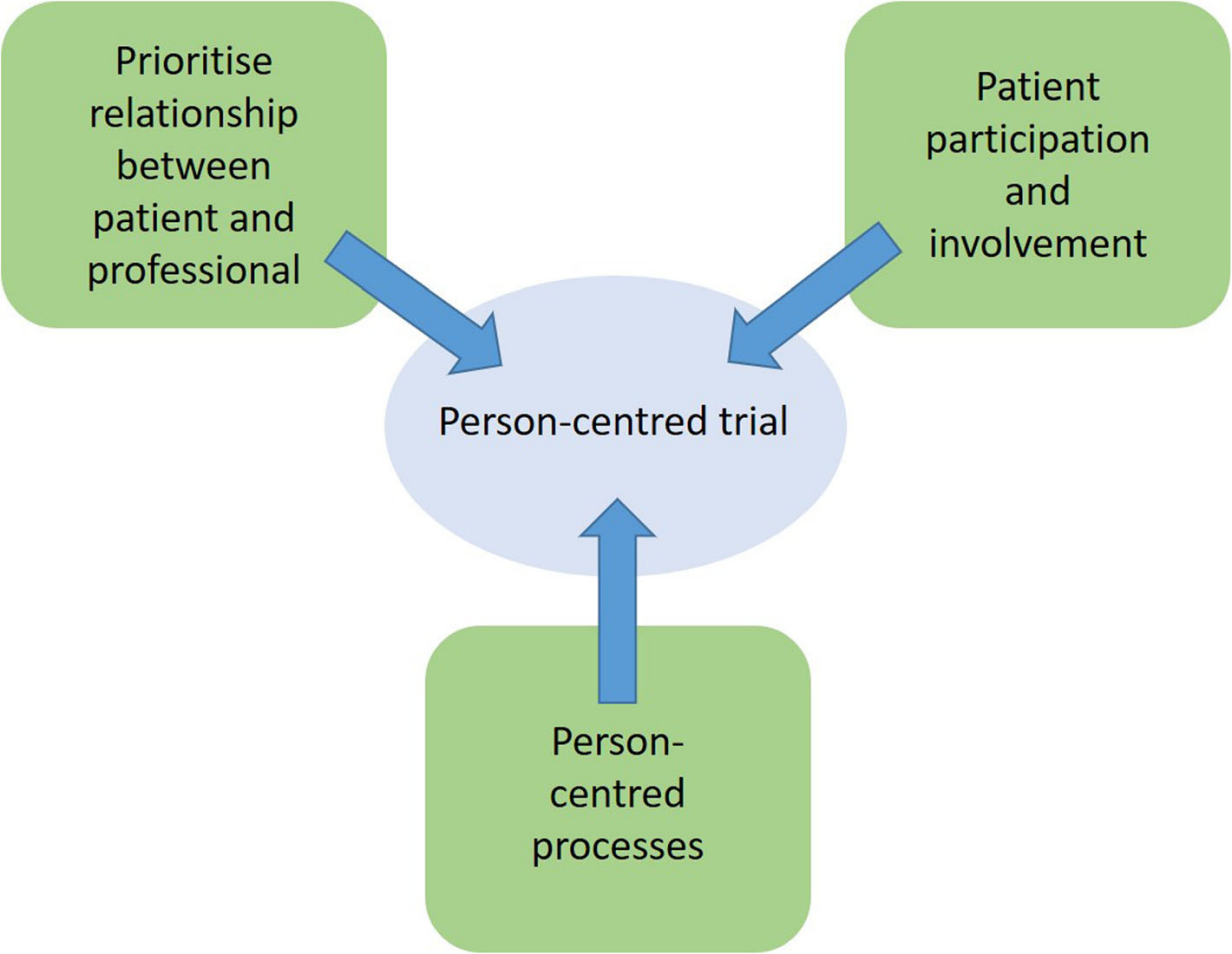
The person-centred trial

On the basis of core concepts of person-centred care described by Kitson et al. [32], and following feedback from PPI representatives, we developed the model of a person-centred trial (Fig. 1). Our study design aimed to put the patient at the centre of the trial and minimise study burden, thereby enabling participants to be actively involved and able to participate. The design focused on developing a genuine relationship between the researcher and participant, with emphasis on continuity. Burden from the trial was minimised by offering home visits and helping participants to complete trial-related questionnaires to ensure a supportive system. PPI contributed to all stages of the trial, from design to analysis, with representatives on the trial management group and the trial steering committee. Trial burden was highlighted as important, and changes were made to the patient information sheet to ensure a clearer explanation of trial processes, including the concept of randomisation.

In-depth interviews were conducted with patients who had taken part in a double-blind, randomised feasibility trial of mirtazapine for chronic or refractory breathlessness. Ethical approval was received from the UK Health Research Authority (16/LO/0091), and the trial was prospectively registered (ISRCTN 32236160). The study is reported in accordance with the Consolidated Criteria for Reporting Qualitative Studies (COREQ) [33].

### Setting

Participants were recruited from three UK sites: King’s College Hospital, Nottingham City Hospital NHS Trust, and Castle Hill Hospital. Potential participants were identified through inpatient clinical teams, multidisciplinary team meetings, hospital clinic lists, and hospital databases. At each site there was a small dedicated research team who were involved in both the recruitment and follow-up data collection across all time points of the trial.

### Study participants and sampling

Those eligible for the feasibility trial were adults with cancer, chronic obstructive pulmonary disease (COPD), interstitial lung disease (ILD), or chronic heart failure (CHF), with a modified Medical Research Council (mMRC) dyspnoea scale grade 3 (‘I stop for breath after walking about 100 yards or after a few minutes on the level’) or 4 (‘I am too breathless to leave the house’ or ‘I am breathless when dressing’), with no current diagnosis of severe depression, and not currently prescribed an antidepressant medication. For full eligibility criteria see Appendix 1. A sampling frame was agreed which included characteristics considered to be important, including gender, diagnosis, trial completion/noncompletion, and age (< 65 years/> 65 years). However, due to the limited pool of participants we decided to take a pragmatic approach and used convenience sampling, offering each trial participant the opportunity to participate in a qualitative interview. Participants were approached by telephone or in person to arrange an interview. All participants provided written informed consent prior to their interview.

### Trial schedule

Patients and carers were approached by their usual clinician and provided with some initial information about the trial. If they were in agreement, they were then contacted by a researcher, who was able to provide more detailed information, including the rationale for doing the study, the trial design, and what it would mean if they agreed to take part in terms of the intervention and study assessments. All members of the research team had training and experience of working with people living with advanced disease. Patients were given a minimum of 24 h to consider the trial and discuss it with friends and family. Participants then provided written informed consent, and a more detailed eligibility assessment followed. After randomisation, the medication was provided along with a diary to complete, details of whom to contact with any questions or concerns, and emergency contact details for out of hours. Participants received 28 days of trial treatment (either oral mirtazapine or placebo capsules). They were assessed face to face on day 0, day 14, and day 28, and via telephone on day 7, day 21, and day 35. Assessments were organised at a time which was convenient for the participant with some flexibility (± 1 day). Participants were offered to be visited at home, and assistance was provided with completing the trial-based questionnaires. Continuity of the researcher was prioritised where possible.

### Data collection

Qualitative interviews were conducted at the end of the trial. Interviews were conducted in a place of the participant’s choosing. This was usually their own home, but some interviews were conducted in hospital. The topic guide (Appendix 2) was developed using existing literature and refined following feedback from PPI representatives and the trial management group [1–6]. The interview schedule included questions about experience of recruitment to the trial, why they had decided to take part, expectations of the trial, and experience of trial processes (taking the trial medication, experience of trial visits, and experience of completing the trial questionnaires). Interviews were digitally audio recorded and transcribed verbatim. A distress protocol was used to minimise the risk of potential harm. All interviews were conducted by one female researcher (NL) with a medical background, who had completed training in in-depth interviewing. Interviews took place between January 2017 and December 2017.

### Analysis

The qualitative interviews were analysed using Braun and Clarke’s framework for thematic analysis [29] using NVivo version 10 software (QSR International UK Ltd., Warrington, UK). Transcripts were read and re-read and then coded inductively for themes relating to reasons to participate in the trial, reasons not to participate in the trial, reasons to remain in the trial and reasons to discontinue the trial. Results were considered in relation to the core elements of person-centred care and our model of the person-centred trial (Fig. 1) [32]. Three transcripts were double-coded by another researcher (SNE), who produced their own coding frame. Areas of agreement and disagreement were then discussed until consensus was achieved.

## Results

The feasibility trial was open to recruitment between August 2016 and November 2017. Each centre was open for a total of 12 months. A total of 409 patients were screened; 150 were eligible, and 64 were randomised. No participants were lost to follow-up. Twelve participants discontinued treatment prior to day 28, five of whom withdrew from data collection. Forty participants (63%) required some help competing the trial questionnaires.

The qualitative interviews were conducted between January 2017 and December 2017. The median time between trial completion and qualitative interview was 83 days (range 1–252). Twenty-two participants were interviewed. Eleven had a diagnosis of COPD, 8 ILD, 2 CHF and 1 lung cancer. The median age was 71 years (range 56–84). Sixteen were male. Twenty had completed the trial, whilst two withdrew due to reported adverse effects of the trial medication. The mean interview duration was 33 min (range 15–104). Despite the use of convenience sampling, variation was achieved, and we interviewed participants from all three research sites, all disease groups, both age and gender categories, with two non-completers also participating in interviews. No trial decliners agreed to complete a qualitative interview (Table 1).

**Table 1:** Characteristics of participants

	Male	Female
ILD		
< 65 years old	1	
> 65 years old	5	3^a^
COPD		
< 65 years old	2	1
> 65 years old	5	1
CHF		
< 65 years old		
> 65 years old	2^a^	
Cancer		
< 65 years old		
> 65 years old	1	1

*Abbreviations: CHF* congestive heart failure, *COPD* chronic obstructive pulmonary disease, *ILD* interstitial lung disease

^a^One did not complete trial

The relationship between patient and professional, potential for benefit, trial processes and the intervention all influenced the decision to participate in the trial. The relationship and continuity with the research team, perceived benefit, and aspects relating to trial processes and the intervention influenced the decision to remain in the trial.

### What influenced people to take part in the trial

#### Approach

The way in which potential participants were approached was important when considering whether to take part in the trial. Many chose to participate because of their relationship with their usual clinician. Being approached by someone familiar appeared to validate the authenticity of the trial. A genuine patient–professional relationship based on open communication, knowledge and skills was valued and made patients more likely to agree to be contacted by a researcher.

*‘It came through when I was at the IPF meeting and my consultant was there that day giving a talk so I figured it was bona fide’. − 1010, female with ILD > 65 years old*

*‘My doctor said ‘well try it, anything’s worth a try’. It’s our GP… we’ve known him for a while… he’s a doctor that listens to you.… He’s very good like’. − 1022, male with COPD > 65 years old*

The initial encounter with the researcher was key. Clear communication of trial-related material established confidence in the research team. Despite some participants having concerns about the expectation that might be placed on them, they felt reassured when the initial assessment was tailored and focused to their individual needs (e.g., by ensuring that the participant did not feel rushed, and helping them to complete the trial questionnaires). Fundamental was the ability of the researcher to assess and meet these individual needs:

*‘The interviewers were very pleasant, very helpful, they explained everything to me, and I agreed to it’. – 1001, male with COPD > 65 years old*

*‘I thought, I hope they’re not going to push me too much … but everything was fine, you know, spot on. They understood my needs. People took the time and they listen to you’. – 1014, male with COPD < 65 years old*

#### Motivations to take part

The possibility of potential benefit was a large contributing factor when deciding whether to participate in the trial. Most commonly participants described hoping for an improvement in symptoms, above all their breathing. Many viewed the trial as an opportunity to have extra input from clinical services, including additional assessments prior to enrolment, regular monitoring throughout the trial, and being seen by a specialist.

*‘I was prepared to try anything that would help with me breathing’. – 1015, female with COPD > 65 years old*

*‘I had a full medical before I started on the course, which was good, it eased my mind’. – 1015, female with COPD > 65 years old*

*‘They just told us that we would be regularly monitored’. – 1010, female with ILD > 65 years old*

*‘It opens doors at the hospitals for you, like I’ve got to see a specialist through it’. – 1022, male with COPD > 65 years old*

For many, living with chronic or refractory breathlessness can be an isolating experience, and therefore the social aspect of participating in the trial was perceived as a potential benefit, with the trial providing an opportunity to meet other people who were in a similar position.

*‘I was gonna gain in that I would be meeting a few more people’. – 1009, male with COPD > 65 years old*

Participants appeared to understand the concept of randomisation and were mostly accepting of the fact that they may not receive the active medication. However, some participants did express concerns about receiving the placebo medication and missing out on a potential benefit from the active medication.

*‘I just sort of tried to take it in my stride, whichever I get, I get, cause there’s not a lot you can do about it’. – 1001, male with ILD > 65 years old*

*‘Only if it wasn’t the drug … then there might not be a chance of it working’. – 1008, male with ILD < 65 years old*

Altruism was also commonly described, and people wanted to participate to help others, regardless of whether they would experience a direct benefit. One man with COPD explained that he did not expect the trial to help him but hoped it might benefit others in the future. Participants also talked about their individual experience of receiving healthcare, often over a number of years, and many felt that the trial was an opportunity to be involved and give something back to the health service. Some people recognised the importance of clinical trials in the context of research and wanted to participate to advance science and help to develop new treatments.

*‘It won’t do me any good but it might help other people in the future, you know. So, my expectations are in the ways that it’ll help other people in the future, you know, by me taking a part in these trials’. – 1014, male with COPD < 65 years old*

*‘I have had some wonderful service from the NHS [National Health Service], and I thought well this is a chance to pay something back by taking part’. – 1004, male with COPD > 65 years old*

*‘People need to know about these things…. If it is going to help then I’ll take part in these trials. To, you know, help, help science’. – 1005, male with ILD > 65 years old*

#### Trial design and the intervention

The trial design was important when deciding whether to participate and attempts to minimise burden were viewed favourably by participants. The opportunity to be visited at home instead of going into hospital was a positive influence and made people more likely to participate in the trial.

*‘I didn’t have to go to the hospital…. You do home visits, and that, that made my mind up even more to do it. Because of the struggling to walk and everything else, so I was more than happy’. – 1003, male with COPD > 65 years old*

The intervention was perceived as simple and low risk, and for some it was important that they could continue other disease-specific medications but still be part of the trial.

*‘The taking of the medication was simple, I didn’t forget it once’. – 1004, male with COPD > 65 years old*

*‘I rang up the hospital and asked, and they said, ‘Yeah, you’ll be ok, ones for your brain and ones for your lungs’. – 1010, female with ILD > 65 years old*

Whilst some participants expressed concerns about taking an antidepressant medication, this was mostly offset by implicit trust in the clinicians and researchers, and a belief that they wouldn’t be given anything which could cause harm.

*‘That was my thought when they first said antidepressant, ‘Oh, do I want to be taking something like that?’ But at the end of the day, they’re not going to do anything that’s going to put you at any risk’. – 1020, male with COPD < 65 years old*

Although we only interviewed people who had participated in the trial, the interviews did highlight some concerns relating to the intervention. One participant who experienced adverse effects and later withdrew from the trial felt that more information could have been provided about the trial medication.

*‘It wasn’t a great deal of information about the actual drug, to be honest’. – 1016, male with heart failure > 65 years old*

### What influenced people to remain in the trial

#### Importance of the relationship and continuity of care

The importance of the relationship between the participant and the researcher was identified across all interviews and was substantial when considering the reasons why people remained in the trial. Attempts by the researcher to minimise burden and ensure a calm environment were recognised and appreciated by participants. The personal attributes of the researcher were also central to remaining in the trial. Participants described the importance of effective communication, being treated with respect, and not feeling rushed during trial visits.

*‘I found the people extremely helpful; nothing was too much trouble. Everything was explained in meticulous detail really, it was so easy, everything was done for you, the drugs were all measured out you had the right number for the right days. All I had to do was wake up and pop the pill, you know. The people were lovely, it was a very, very rewarding experience in a lot of ways’. – 1020, male with COPD < 65 years old*

*‘Like [the research nurse] said, if there’s any problems and you can’t make it, just give us a ring or anything like that, there’s no, you must arrive or that sort of thing. And it’s a relaxing place, when you go there, there’s no hustle and bustle’. – 1013, male with COPD > 65 years old*

*‘The [research nurses] are absolutely brilliant, and that does make a difference, you know that you’re going to walk in.… They explain things so well don’t they, and they’re so patient and you know’. – 1012, male with ILD > 65 years old*

*‘They ask you a question, but they listen to you, they didn’t jump in and try to answer for you. I was number one, you know what I mean’. – 1014, male with COPD < 65 years old*

Continuity was important and enabled participants to build up a relationship with the research team. One participant explained that while they did not always see the same member of the research team, someone they had met before always made an effort to come and say hello when they arrived.

*‘I’d go in and sit down, they’d maybe make me a cup of tea if I was waiting and whatever, then they’d come through. It wasn’t always the same person, but [research nurse] would pop in and say hello and she’d say so-and-so’s seeing you today’. – 1020, male with COPD < 65 years old*

In contrast, not being given clear trial-related information and feeling rushed by members of the research team was reported by one participant who chose to withdraw from the trial. While the participant chose to withdraw due to adverse effects of the trial medication, these other factors may have contributed to this decision.

*‘It was a bit rushed wasn’t it’? – 1016, male with heart failure > 65 years old*

#### Perceived benefits

Perceived benefits from the trial medication motivated people to remain in the trial. Participants described improved breathing but also beneficial effects on sleep, fatigue and appetite, which for some led to increased confidence and an ability to be more active. Participants also perceived the regular monitoring they received during the trial to be beneficial and describing feeling ‘taken care of’ during the trial period.

*‘Everything was so much better. I would sleep better, so if I sleep better that means by breathing is better when I wake up in the morning, which it never was before. Everything has just changed for the better’. – 1003, male with COPD > 65 years old*

*‘The follow-up has been very good. I was seen at weekly intervals to see how things were progressing, and if there were any problems, so I felt I was being taken care of in terms of the trial’. – 1017, male with ILD > 65 years old*

The social aspect was an additional benefit for many participants and provided an interruption to an otherwise sometimes isolating existence. This was described by participants visited at home but also by those who were reviewed in the clinical trials unit.

*‘I quite enjoyed the experience of having somebody to come in and talk to me’. – 1001, male with ILD > 65 years old, visited at home*

*‘They could’ve come to my home, but I prefer to come here ’cause it gets me out the house for an hour or two…. It’s nice just to come somewhere and, as I say, meet different people, see different people, which is half the battle when you, you know’. – 1014, male with COPD < 65 years old, attended the trials unit*

It was important that participants felt actively involved and as though they were contributing to the trial. Knowing that the trial may benefit patients in the future, as well as providing an opportunity for individuals to give back were motivating factors for completing the trial. Several participants described how they found the trial process rewarding on an individual level.

*‘I just felt as though I was doing some good. It was personally rewarding for me, because I felt as though I was contributing, you know’. – 1020, male with COPD < 65 years old*

#### Trial processes and the intervention

Aspects relating to the trial design and intervention were also important when considering the reasons why participants remained in the trial. The offer of home visits reduced the burden of participating, and while participants described the questionnaires as straightforward, they were grateful when help was provided.

*‘Being at home was perfect, they were always on time, and prompt. Oh the home visits are quite good you know. Saved me a lot of bother not going to the hospital’. – 1002, male with COPD > 65 years old*

*‘If there were any problems, then they would run me through the questions’. – 1020, male with COPD < 65 years old*

The intervention was simple and well tolerated, and participants found the chart provided a useful reminder. Trial duration was also important, with a shorter duration felt to be more manageable.

*‘It was tablets and I took them every day as I was asked to, um, we made a note of them in a chart to make sure I had taken them, it was no problem at all’. – 1001, male with ILD > 65 years old*

*‘I thought that as it was also only over a 28-day period, I thought, yeah, I’d, I’d be quite happy to try’. – 1010, female with ILD > 65 years old*

Adverse effects of the intervention were an important influence for participants discontinuing the trial and were reported by both participants who were interviewed after withdrawing from the trial.

*‘I just sat up in bed looking at the tablets and thinking, should I chance it tonight or not, because I knew how I might feel a bit groggy the next day, so it put you off taking the tablet’. – 1019, female with ILD > 65 years old*

## Discussion

This study identifies important considerations which may influence recruitment and retention in clinical trials. We found that the relationship between patient and professional, potential for benefit, trial processes and the intervention all influenced the decision to participate in the trial. The relationship with the research team and continuity, perceived benefit, and aspects relating to trial processes and the intervention influenced the decision to remain in the trial. In this trial recruitment targets were met and attrition levels were low, suggesting that a person-centred approach can support successful recruitment and retention.

What influences potential participants to take part in a clinical trial (or not) is recognised to be a complex multifactorial process [34–39]. In this study we found that the initial approach by both clinician and researcher was key in developing a genuine relationship built on trust, a concept which has been identified as important when deciding whether to participate in a clinical trial [34, 35, 40]. In this study participants described the potential benefit to self and others as a motivating factor, comparable to the findings of previous qualitative research conducted in the palliative care setting [37]. While concerns about randomisation and the potential for side effects can be deterrents to participating in a clinical trial [36], this was not a major influencing factor for the participants we interviewed. The trial design was important, and attempts to minimise burden were viewed favourably by participants. This is an important consideration as missing data in trials has been shown to increase with the number of questionnaires/tests [17].

In this study the relationship between the patient and professional was crucial, and particularly important when considering what influenced people to remain in the trial. Feeling listened to, being treated with respect, and having their needs understood were important influences supporting retention. The continuity of the research team was also important and enabled participants to build up a trusting relationship over the trial duration; one participant referred to this as ‘feeling like part of the family’. In addition, participants praised the research team for the extra time taken during trial visits. This ensured that individuals did not feel rushed and allowed assessments to be completed in the participant’s own time. These findings have implications for the set-up of research teams across trials. While our results highlight the importance of developing a genuine patient–professional relationship, this needs to be balanced so that patients do not feel coerced to take part or remain in a trial. Training and the use of standard operating procedures are also crucial to ensure that assistance with questionnaires is applied in a consistent manner. Although there are often concerns about including people with advanced disease in studies, research suggests that those living with advanced disease want the opportunity to be involved in research and report it to be a positive experience from which they benefit [41].

The trial design and trial processes were also important considerations, particularly for trial retention. It has been suggested that an individualised design, based on individual capabilities, which enables participation alongside the other challenges in life, may have a positive impact on trial retention [19]. We applied a person-centred approach by providing clear trial-related information, offering home visits, involving the carer, and assisting with trial-related questionnaires. PPI was crucial, and feedback from representatives ensured that that the trial worked around the patient and not the other way around.

The results of this study have important implications for policy and funding. In our trial, a small, dedicated research team facilitated a genuine relationship based on open communication, knowledge and the perceived skill set of the researcher. Home visits and spending time with the participant, often helping them to complete trial questionnaires (63% of participants in this trial), was important. Time and resource constraints have been acknowledged as limitations in other studies, and if we are to improve retention within trials, we need to ensure that funding allows adequate resource allocation to spend time supporting participants with trial processes [26]. While our study suggests a benefit to having the same researchers working across all stages of a trial, current funding models in the United Kingdom focus specifically on recruitment and not on retention, and therefore the funding for follow-up often needs to be pooled from other budgets [42]. In practice, continuity of research staff is not a commonly reported outcome, and so it is difficult to know the impact of this across different specialties and for larger trials. To ensure that the same researchers are able to work across trials, funding models need to be revised to rebalance of emphasis of recruitment and retention [43].

It is important to acknowledge that the researchers in our trial all had training and experience in working with people living with advanced disease. Participants valued the personal attributes of the professional, a quality which has been identified as critical in person-centred care [32]. Characteristics which have previously been identified as important for palliative care professionals include interpersonal skills, a willingness to listen, being someone the patient feels able to talk to, demonstrating an interest in knowing patients as people, and recognising that patients may need to feel in control [32, 44]. Therefore, the attributes of professionals delivering person-centred care and palliative care are closely aligned [45]. Increased opportunities for the training of research staff has been highlighted as important if we are to improve retention in clinical trials in the future [20, 43].

### How can person-centred care be applied to clinical trials in practice?

**Fig. 2:**
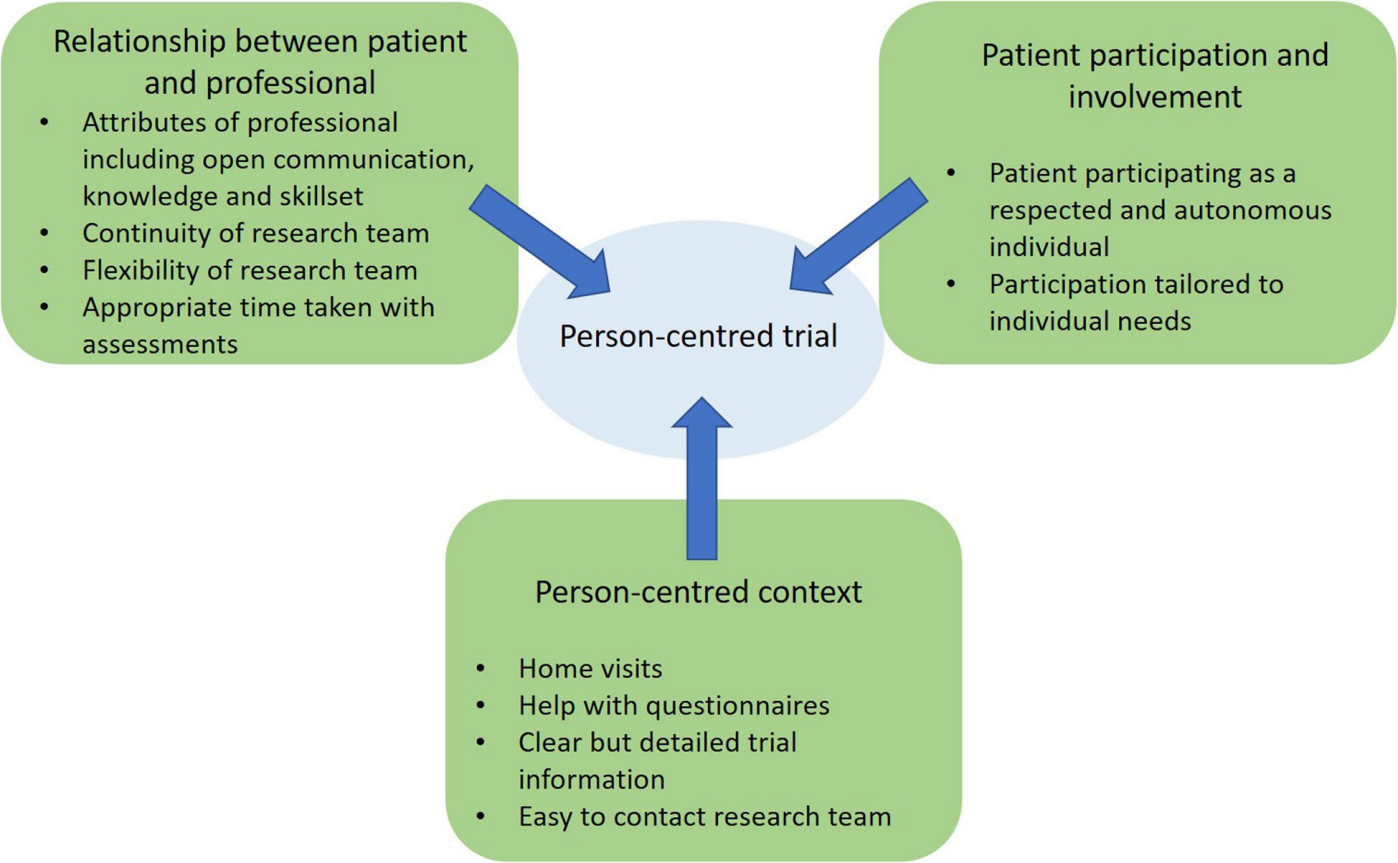
The person-centred trial in practice

To improve retention, clinical trials need to be individualised, with processes in line with individual capabilities, and considered alongside the other challenges in life [19]. We propose that implementing a person-centred approach can support recruitment and retention. Our model focuses on three key areas: development of a genuine relationship between the participant and professional, enabling participation, and ensuring that trial processes are person-centred (Fig. 2). Education and training can help to provide professionals with the required knowledge and skill set and ensure that trial assessments are tailored to the holistic needs of the individual. Continuity of the research team provides an opportunity for the researcher and participant to build a genuine relationship during the trial period. Person-centred trial processes such as home visits and helping participants to complete trial-related questionnaires help to minimise the burden for participants.

### Strengths and limitations

To our knowledge, this is the first study to consider what influences people to take part and, more importantly, remain in a clinical trial in the context of advanced disease. The study used in-depth interviews, and despite the use of convenience sampling, achieved variation with participants across all characteristics identified to be important. While a single researcher conducted all of the interviews, interpretation bias was minimised by use of a reflexive diary, double-coding of a subset of transcripts, and discussion of findings within the research team.

The study was limited by one female researcher (NL) with a medical background conducting all of the interviews. In addition, some of the interviewees had met this researcher during the feasibility trial, thereby increasing the risk of social desirability bias, and participants may have been reluctant to offer criticisms about the trial intervention and/or processes. The time period between the trial ending and a qualitative interview being conducted varied, and this may have increased the risk of recall bias in the qualitative interviews. Some interviews were conducted with a carer present, which may have impacted the answers given. Although we achieved a varied sample of trial participants, we only interviewed two participants who did not complete the trial, and we were not able to interview anyone who declined to participate in the trial.

The trial itself was of short duration with an arguably simple intervention and may therefore be perceived as easier in terms of recruitment and retention than a longer trial or one of a complex intervention. However, challenges with recruitment (in part due to eligibility) and high attrition levels have previously been demonstrated in short-duration drug trials conducted in people with advanced disease [5]. Sixteen of the interviews were conducted with male patients, which is reflective of the main trial participants. This is similar to other trials [46] and may reflect the fact that chronic lung disease has previously been considered to be a condition predominantly affecting men [47]. It has recently been acknowledged, however, that women remain underrepresented in chronic lung disease trials, and this should be addressed in future research [48]. With an aging population, an increasing number of people are living with chronic and complex conditions and multimorbidity. The findings of our study are therefore relevant and important for clinical trials in the future.

## Conclusions

This study identifies important considerations which influenced the decision to participate and remain in a feasibility trial of mirtazapine for chronic or refractory breathlessness. Results should be considered within the context of the existing literature, which suggests an increasing role for a person-centred approach in trials. PPI can help to identify how aspects of a trial can be more person-centred and should be incorporated at all stages of trial design. We propose that prioritisation of the relationship between the patient and the professional, ensuring the trial design is as person-centred as possible, and enabling people to participate with processes in line with individual capabilities may improve recruitment and retention in clinical trials in advanced disease. The results of this study have potential implications for the future funding of trials and highlight the importance of having a dedicated research team who are able to build a genuine relationship with participants throughout the duration of a trial. Our model of the person-centred trial should be considered when designing a clinical trial, ideally at the prefunding stage and involving PPI representatives across all stages of trial development and analysis. Future work should aim to evaluate the application of a person-centred approach to clinical trials in different settings.

## Data Availability

Requests for data should be made to the corresponding author.

## Appendix 1

### Full eligibility criteria

*Inclusion criteria:*

1. Male or female aged ≥ 18 years old
2. Diagnosed with cancer, COPD, ILD or chronic heart failure (New York Heart Association class III or IV)
3. Breathlessness severity: mMRC dyspnoea scale grade 3 or 4
4. Receiving optimal treatment of the underlying condition in the opinion of the identifying clinician
5. Management of the underlying condition has remained unchanged for the previous 1 week
6. Reversible causes of breathlessness optimally treated in the opinion of the identifying clinician
7. Expected prognosis ≥ 2 months
8. If female and of child-bearing potential, agrees to use adequate contraception
9. Able to complete questionnaires and trial assessments
10. Able to provide written informed consent

*Exclusion criteria:*

1. Existing antidepressant use
2. Known contraindication to mirtazapine
3. Hypersensitivity to the active substance or to any of the components of the mirtazapine or placebo (e.g., lactose intolerance)
4. Australia modified Karnofsky Performance Status score ≤ 40
5. Pregnant or breast-feeding women
6. Patients with acute cardiac events within 3 months of randomisation (myocardial infarction, unstable angina pectoris or significant cardiac conduction disturbance)
7. Patients with known hepatic impairment
8. Patients with known renal impairment
9. Patients with uncontrolled blood pressure
10. Patients with uncontrolled diabetes mellitus
11. Patients with uncontrolled seizures, epilepsy or organic brain syndrome
12. Patients with severe depression or suicidal thoughts
13. Patients with a history of psychotic illness (schizophrenia, bipolar disorder, mania, hypomania or other psychotic disturbances)

## Appendix 2

### Topic guide

Topic guide

You have recently taken part in a study called Better B. I would like to talk to you to understand your experience of taking part, what you expected, and what it was like.

If you want to stop the interview at any point, let me know. You don’t need to give a reason, and your clinical care won’t be affected. Everything you say will be kept confidential.

Do you have any questions before we begin?

Introduction/Better-B

What did you understand about the study?

What was your experience of taking part?

*Prompt: Can you tell me a bit about that?*

Recruitment/joining the study

How were you asked to take part in the study?

What was that like?

*Prompt: Who spoke to you? What were you told? Where were you at the time?*

What were your expectations?

Why did you decide to take part?

*Prompt: What specifically did you want to see improved? What changes were you hoping for?*

Trial processes/taking part

What did you understand about the treatment you received?

*Prompt: What did you think about taking an antidepressant medication? What do you understand about a placebo drug/randomisation?*

How did you find taking the medication?

*Probe: Did you have any difficulties? How did you manage with your other medications? (dosette box/blister pack/diary as reminder).*

How did you like being visited at home?

Would you have preferred to have been seen somewhere else?

How did you find it completing the questionnaires?

*Probe: What did you think about the questions we asked? Do you think they were the right questions? Did they capture what is important to you?*

Would anything have made it easier to take part?

*Probe: What were the downsides to taking part?*

Change

Tell me in what ways the drug changed how you felt?

*Prompt: Did you notice any change in your breathing, sleep, appetite, drowsiness?*

What did you hope would change?

For you, what would be the most important change?

Were there any changes you had not expected?

Closing section

Is there anything else that you think is important for me to know?

Is there anything that has worried you during the course of this conversation?

Is there anything else you would like to talk about?

## Abbreviations

BETTER-B: BETter TreatmEnts for Refractory Breathlessness
CHF: Chronic heart failure
COPD: Chronic obstructive pulmonary disease
COREQ: Consolidated Criteria for Reporting Qualitative Studies
ILD: Interstitial lung disease
mMRC: Modified Medical Research Council dyspnoea scale
NIHR: National Institute for Health Research
PPI: Patient and public involvement
RCT: Randomised controlled trial

## References

[1] Walters SJ, Dos Anjos Henriques-Cadby IB, Bortolami O, Flight L, Hind D, Jacques RM (2017). Recruitment and retention of participants in randomised controlled trials: a review of trials funded and published by the United Kingdom Health Technology Assessment Programme. BMJ Open.

[2] Bouca-Machado R, Rosario M, Alarcao J, Correia-Guedes L, Abreu D, Ferreira JJ (2017). Clinical trials in palliative care: a systematic review of their methodological characteristics and of the quality of their reporting. BMC Palliat Care.

[3] Boland J, Currow DC, Wilcock A, Tieman J, Hussain JA, Pitsillides C (2015). A systematic review of strategies used to increase recruitment of people with cancer or organ failure into clinical trials: implications for palliative care research. J Pain Symptom Manage.

[4] Solheim TS, Laird BJA, Balstad TR, Stene GB, Bye A, Johns N (2017). A randomized phase II feasibility trial of a multimodal intervention for the management of cachexia in lung and pancreatic cancer. J Cachexia Sarcopenia Muscle.

[5] Fallon M, Hoskin PJ, Colvin LA, Fleetwood-Walker SM, Adamson D, Byrne A (2016). Randomized double-blind trial of pregabalin versus placebo in conjunction with palliative radiotherapy for cancer-induced bone pain. J Clin Oncol.

[6] Stone PC, Gwilliam B, Keeley V, Todd C, Kelly LC, Barclay S (2013). Factors affecting recruitment to an observational multicentre palliative care study. BMJ Support Palliat Care.

[7] Rinck GC, van den Bos GA, Kleijnen J, de Haes HJ, Schade E, Veenhof CH (1997). Methodologic issues in effectiveness research on palliative cancer care: a systematic review. J Clin Oncol.

[8] Hanson LC, Bull J, Wessell K, Massie L, Bennett RE, Kutner JS (2014). Strategies to support recruitment of patients with life-limiting illness for research: the Palliative Care Research Cooperative Group. J Pain Symptom Manage.

[9] Ewing G, Rogers M, Barclay S, McCabe J, Martin A, Todd C (2004). Recruiting patients into a primary care based study of palliative care: why is it so difficult?. Palliat Med.

[10] McWhinney IR, Bass MJ, Donner A (1994). Evaluation of a palliative care service: problems and pitfalls. BMJ.

[11] Jordhoy MS, Kaasa S, Fayers P, Ovreness T, Underland G, Ahlner-Elmqvist M (1999). Challenges in palliative care research; recruitment, attrition and compliance: experience from a randomized controlled trial. Palliat Med.

[12] Bajwah S, Ross JR, Peacock JL, Higginson IJ, Wells AU, Patel AS (2013). Interventions to improve symptoms and quality of life of patients with fibrotic interstitial lung disease: a systematic review of the literature. Thorax.

[13] Kearney A, Daykin A, Shaw ARG, Lane AJ, Blazeby JM, Clarke M (2017). Identifying research priorities for effective retention strategies in clinical trials. Trials.

[14] Tudur Smith C, Hickey H, Clarke M, Blazeby J, Williamson P (2014). The trials methodological research agenda: results from a priority setting exercise. Trials.

[15] Brunsdon D, Biesty L, Brocklehurst P, Brueton V, Devane D, Elliott J (2019). What are the most important unanswered research questions in trial retention? A James Lind Alliance Priority Setting Partnership: the PRioRiTy II (Prioritising Retention in Randomised Trials) study. Trials.

[16] Hui D, Glitza I, Chisholm G, Yennu S, Bruera E (2013). Attrition rates, reasons, and predictive factors in supportive care and palliative oncology clinical trials. Cancer.

[17] Hussain JA, White IR, Langan D, Johnson MJ, Currow DC, Torgerson DJ (2016). Missing data in randomized controlled trials testing palliative interventions pose a significant risk of bias and loss of power: a systematic review and meta-analyses. J Clin Epidemiol.

[18] Hussain JA, Bland M, Langan D, Johnson MJ, Currow DC, White IR (2017). Quality of missing data reporting and handling in palliative care trials demonstrates that further development of the CONSORT statement is required: a systematic review. J Clin Epidemiol.

[19] Skea ZC, Newlands R, Gillies K (2019). Exploring non-retention in clinical trials: a meta-ethnographic synthesis of studies reporting participant reasons for drop out. BMJ Open.

[20] Bower P, Brueton V, Gamble C, Treweek S, Smith CT, Young B (2014). Interventions to improve recruitment and retention in clinical trials: a survey and workshop to assess current practice and future priorities. Trials.

[21] Mullins CD, Vandigo J, Zheng Z, Wicks P (2014). Patient-centeredness in the design of clinical trials. Value Health.

[22] Crocker JC, Ricci-Cabello I, Parker A, Hirst JA, Chant A, Petit-Zeman S (2018). Impact of patient and public involvement on enrolment and retention in clinical trials: systematic review and meta-analysis. BMJ.

[23] Sidani S, Fox M, Collins L (2017). Towards patient-centered clinical trial designs. Eur J Pers Cent Healthc.

[24] Bagley HJ, Short H, Harman NL, Hickey HR, Gamble CL, Woolfall K (2016). A patient and public involvement (PPI) toolkit for meaningful and flexible involvement in clinical trials - a work in progress. Res Involv Engagem.

[25] Nilsen ES, Myrhaug HT, Johansen M, Oliver S, Oxman AD (2006). Methods of consumer involvement in developing healthcare policy and research, clinical practice guidelines and patient information material. Cochrane Database Syst Rev.

[26] Chhatre S, Jefferson A, Cook R, Meeker CR, Kim JH, Hartz KM (2018). Patient-centered recruitment and retention for a randomized controlled study. Trials.

[27] Higginson IJ (2016). Research challenges in palliative and end of life care. BMJ Support Palliat Care.

[28] Visser C, Hadley G, Wee B (2015). Reality of evidence-based practice in palliative care. Cancer Biol Med.

[29] Kane PM, Murtagh FE, Ryan K, Mahon NG, McAdam B, McQuillan R (2015). The gap between policy and practice: a systematic review of patient-centred care interventions in chronic heart failure. Heart Fail Rev.

[30] Burton CD, Entwistle VA, Elliott AM, Krucien N, Porteous T, Ryan M (2017). The value of different aspects of person-centred care: a series of discrete choice experiments in people with long-term conditions. BMJ Open.

[31] Chenoweth L, Forbes I, Fleming R, King MT, Stein-Parbury J, Luscombe G (2014). PerCEN: a cluster randomized controlled trial of person-centered residential care and environment for people with dementia. Int Psychogeriatr.

[32] Kitson A, Marshall A, Bassett K, Zeitz K (2013). What are the core elements of patient-centred care? A narrative review and synthesis of the literature from health policy, medicine and nursing. J Adv Nurs.

[33] Tong A, Sainsbury P, Craig J (2007). Consolidated Criteria for Reporting Qualitative Research (COREQ): a 32-item checklist for interviews and focus groups. Int J Qual Health Care.

[34] Biedrzycki BA (2010). Decision making for cancer clinical trial participation: a systematic review. Oncol Nurs Forum.

[35] Biedrzycki BA (2011). Factors and outcomes of decision making for cancer clinical trial participation. Oncol Nurs Forum.

[36] White CD, Hardy JR, Gilshenan KS, Charles MA, Pinkerton CR (2008). Randomised controlled trials of palliative care – a survey of the views of advanced cancer patients and their relatives. Eur J Cancer.

[37] Gysels M, Shipman C, Higginson IJ (2008). “I will do it if it will help others”: motivations among patients taking part in qualitative studies in palliative care. J Pain Symptom Manage.

[38] Ulrich CM, Ratcliffe SJ, Wallen GR, Zhou QP, Knafl K, Grady C (2016). Cancer clinical trial participants’ assessment of risk and benefit. AJOB Empir Bioeth..

[39] Bowling A, Ebrahim S (2001). Measuring patients’ preferences for treatment and perceptions of risk. Qual Health Care..

[40] Bayly J, Edwards BM, Peat N, Warwick G, Hennig IM, Arora A (2018). Developing an integrated rehabilitation model for thoracic cancer services: views of patients, informal carers and clinicians. Pilot Feasibility Stud.

[41] Gysels MH, Evans C, Higginson IJ (2012). Patient, caregiver, health professional and researcher views and experiences of participating in research at the end of life: a critical interpretive synthesis of the literature. BMC Med Res Methodol.

[42] Department of Health. Attributing the costs of health and social care Research & Development (AcoRD). 4 May 2012. https://www.gov.uk/government/news/attributing-the-costs-of-health-social-care-research-development-acord. Accessed 12 Feb 2020.

[43] Daykin A, Clement C, Gamble C, Kearney A, Blazeby J, Clarke M (2018). ‘Recruitment, recruitment, recruitment’ – the need for more focus on retention: a qualitative study of five trials. Trials.

[44] Johnston B, Smith LN (2006). Nurses’ and patients’ perceptions of expert palliative nursing care. J Adv Nurs.

[45] Saunders DC (2001). Social work and palliative care—the early history. Br J Soc Work.

[46] Currow DC, Ekstrom M, Louw S, Hill J, Fazekas B, Clark K (2019). Sertraline in symptomatic chronic breathlessness: a double blind, randomised trial. Eur Respir J.

[47] Barnes PJ (2016). Sex differences in chronic obstructive pulmonary disease mechanisms [editorial]. Am J Respir Crit Care Med..

[48] Gut-Gobert C, Cavaillès A, Dixmier A, Guillot S, Jouneau S, Leroyer C (2019). Women and COPD: do we need more evidence?. Eur Respir Rev.

